# A rare case of bilateral cervical vagal neurofibromas: role of high-resolution ultrasound

**DOI:** 10.1186/s12883-017-0806-5

**Published:** 2017-02-06

**Authors:** Bin Liu, Yuanding Zhang, Lili Zhang, Fan Zhang, Hongyu Li, Shuang Li, Yafang Liu, Jie Du, Lirong Zhao

**Affiliations:** 1 Department of Hand Surgery, the First Bethune Hospital of Jilin University, Changchun, 130021 China; 2 Department of Otolaryngology, the First Bethune Hospital of Jilin University, Changchun, 130021 China; 3 Department of Electrical Diagnosis, the First Bethune Hospital of Jilin University, No. 3302, Jilin Road, Changchun, 130021 China; 4 Department of Hand Surgery, China-Japan Union Hospital of Jilin University, Changchun, 130033 China; 5 Department of Pathology, the First Bethune Hospital of Jilin University, Changchun, 130021 China

**Keywords:** Vagal neurofibroma, High-resolution ultrasonography, Neurofibromatosis Type 1

## Abstract

**Background:**

Neurofibromas originating from vagus nerves are rarely reported in the literature. In particular, plexiform neurofibromas of the bilateral cervical vagus nerve are extremely rare.

**Case presentation:**

A 21-year-old female presented with a 2-year history of swelling on the right side of her neck. Physical examination revealed a soft-tissue mass on the right side of her neck. Ultrasonography (US) and magnetic resonance (MR) imaging showed a tumor centered in the right carotid sheath between the internal jugular vein and the common carotid artery. In addition, a similar nodular mass in the left carotid sheath was detected on US. The right mass was surgically resected; histopathological examination revealed a neurofibroma.

**Conclusions:**

US can be a valuable method for preoperative evaluation of cervical mass, as it is capable of displaying the vagus nerve and provides sufficient diagnostic information. The cervical vagal neurofibroma can manifest as solitary or multifocal lesion. Bilateral neurofibromas are usually associated with neurofibromatosis type 1 (NF1). Early diagnosis and prompt surgical treatment should be considered.

## Background

Neurofibromatosis type 1 (NF-1) is an autosomal dominant genetic disease, caused by NF1 gene mutations at chromosome 17q11.2 [1]. The condition is characterized by multiple skin lesions such as café-au-lait macules and neurofibromas growing along the parent nerves. Bilateral cervical vagal neurofibromas are extremely rare among patients with neurofibromatosis type 1. To our knowledge, this is the first report of bilateral cervical neurofibromas originating from the vagal nerves. Plexiform neurofibromas represent a special variant of NF-1 in which neurofibromas can arise from multiple nerves as bulging and deforming masses. Herein, we report a rare case of NF-1 with bilateral plexiform neurofibromas arising from cervical vagus nerves.

## Case presentation

**Fig. 1:**
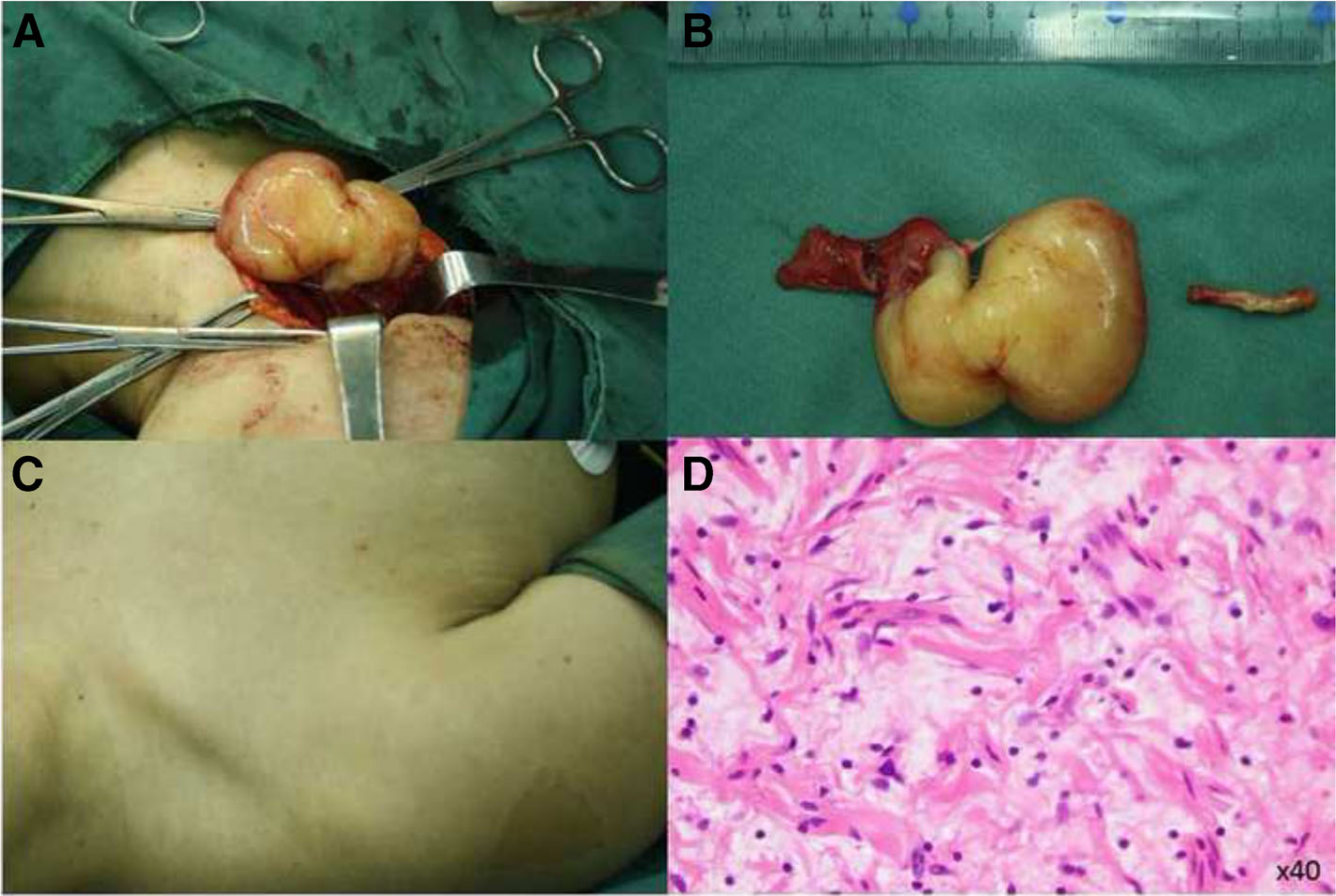
**a** Intraoperative findings demonstrating that the tumor originates from the vagus nerve. **b** After the mass was removed, we cut the affected distal vagus nerve. **c** Physical examination revealing “cafe-au-lait” spots. **d** Histopathological examination showing spindle-shaped cells with narrow nucleus interspersed with thick bands of collagen (hematoxylin and eosin staining; magnification × 40)

A 21-year-old woman presented with a 2-year history of focal swelling on the right side of her neck. She did not have any other significant symptoms. Past medical history was unremarkable and she denied any family history of similar lesions or other skin lesions. Physical examination revealed a 7 cm × 5 cm soft-tissue mass on the right side of her neck, and widespread cafe-au-lait spots on her body (Fig. 1c). The mass had smooth surface and was non-pulsatile; it had mobility in lateral direction but not in vertical direction. No hoarseness of voice or change in heart rate was observed on compression of the mass.

**Fig. 2:**
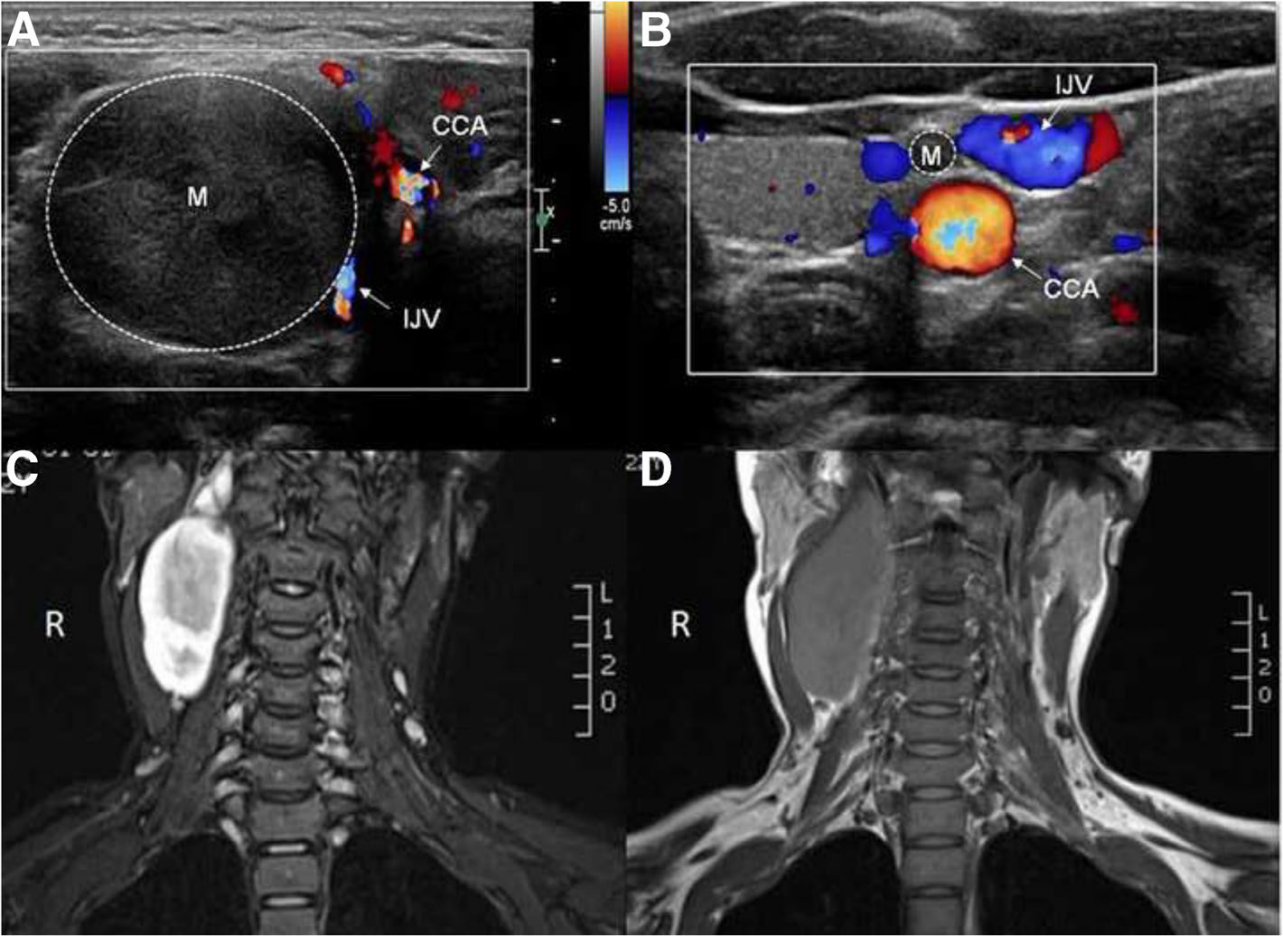
**a** Axial sonogram (5–12-MHz) showing a hypoechoic mass (M, dash line) centered on the right carotid sheath, between the internal jugular vein (IJA) and the common carotid artery (CCA). **b** Axial sonogram (5–12-MHz) showing a hypoechoic mass (M, dash line), vagus nerve (arrow) and common carotid artery (CCA). **c** The coronal T2-weighted MR imaging shows a 7.7 cm × 4.1 cm × 3.0 cm well-defined hyperintense mass adjacent to the right internal carotid artery. **d** The right tumor appeared hypointense on coronal T1-weighted MR imaging

Her neurological examinations were otherwise normal. Magnetic resonance (MR) imaging with a 1.5 TMR scanner (Skyra, Siemens Medical Solutions, Erlangen, Germany) showed a 7.7 cm × 4.1 cm × 3.0 cm well-defined mass adjacent to the right internal carotid artery (Fig. 2c and d). Ultrasonography (US) performed with a linear probe (Philips Healthcare IU22, Bothell, WA, USA), revealed an almost elliptical, well-circumscribed heterogeneously hypoechoic mass centered on the right carotid sheath between the internal jugular vein and the common carotid artery (Fig. 2a). On grayscale images, the mass appeared heterogeneously hypoechoic with no evidence of calcification, cystic change or internal necrosis. Color Doppler US showed strip-like low-velocity arterial flows within the mass. Furthermore, both the proximal and distal ends of the mass were in continuity with a thick cord like structure which possibly represented the vagus nerve. The left carotid sheath also showed an elongated homogenously hypoechoic nodular mass, and both the proximal and distal ends of the mass were seen in continuity with vagus nerve (Fig. 2b). Homogeneous mass lesions from right thigh intermuscular space and surface of left gastrocnemius also were detected on US. Flexible laryngoscopy showed no evidence of vocal cord paralysis.

A right radical neck dissection was performed. Intraoperatively, the tumor was confirmed to originate from the vagus nerve (Fig. 1a); no adhesions to the adjacent common carotid artery or internal jugular vein were noted. Stimulation of the mass did not induce any obvious electrocardiogram abnormalities. The tumor extended up to the jugular foramen and down to the thyroid cartilage. The gross tumor was totally resected from the proximal and distal vagus nerve. After the mass was removed, we found that the distal vagus nerve was also infiltrated, and therefore we cut the affected distal vagus nerve further (Fig. 1b).

**Fig. 3:**
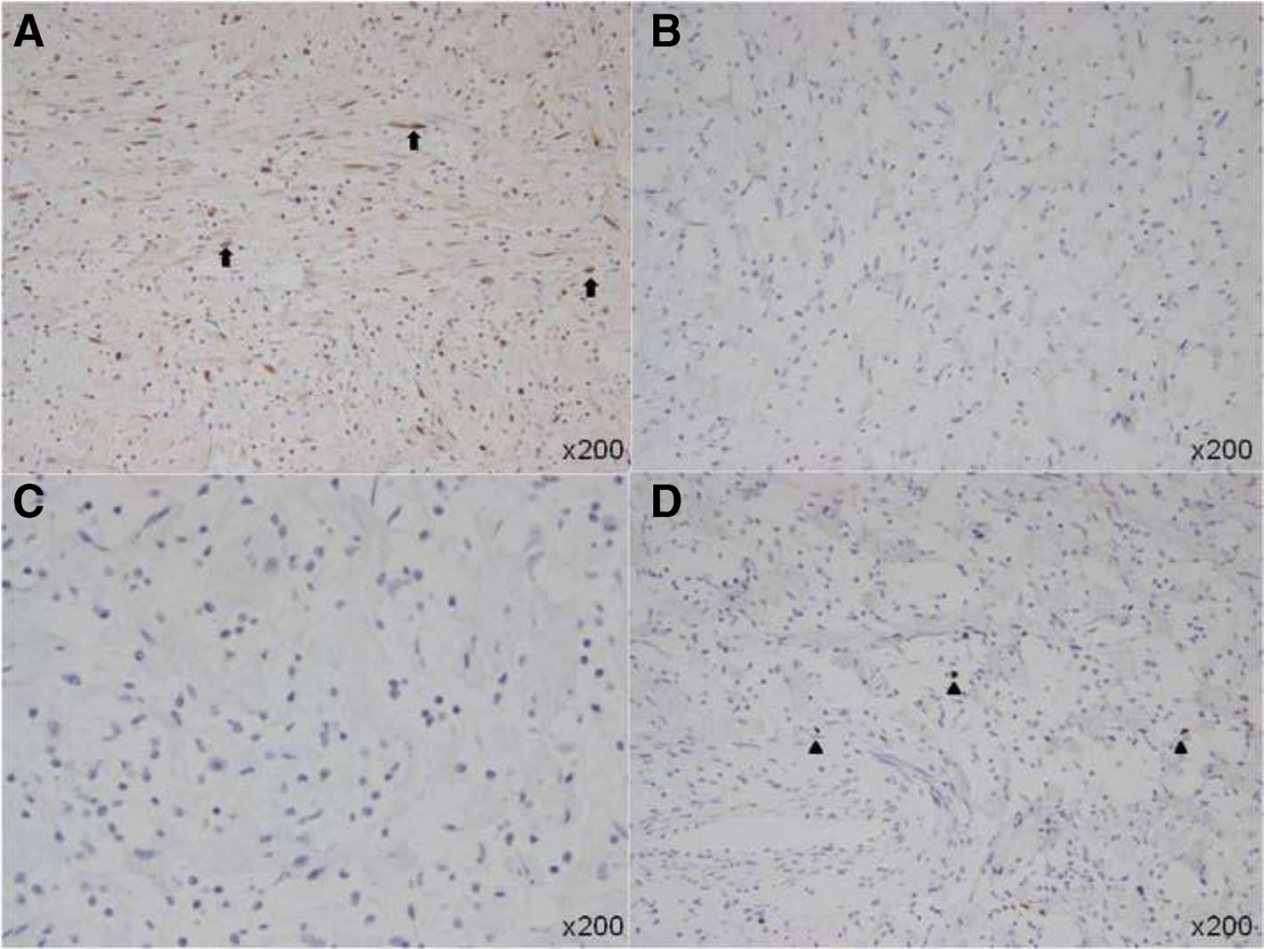
Immunohistochemical staining (original magnification × 200) of NF1-associated neurofibroma showed positivity for S-100 protein (**a**, arrows), while GFAP (**b**) and Olig-2 (**c**) were negative. The Ki-67 labeling index (**d**, arrowheads) was low (<2%)

The pathological examination was consistent with the diagnosis of neurofibroma (Fig. 1d). Immunohistochemical staining showed positivity for S-100 protein, while glial fibrillary acidic portein (GFAP) and oligodendrocyte transcription factor-2 (Olig-2) were negative. The Ki-67 labeling index was low (<2%) (Fig. 3a, b, c and d). Considering that the mass on the left side of her neck was still small, no surgery was performed. However, both the proximal and distal ends of the mass were in continuity with the left vagus nerve, and the peritumoral fibers in the left vagus nerve were also thickened. Combined with the cafe-au-lait spots and multifocal tumors, a diagnosis of NF-1 was established [2]. Postoperatively, there were no cardiac complications, nevertheless a laryngoscopy revealed the right vocal cord palsy. A regular ultrasonographic follow-up was scheduled. During a follow-up period of 8 months, no recurrence or progression of the tumors was observed.

## Discussion

Bilateral vagal nerve schwannomas are more generally found in the context of NF-2. Neurofibromas that arise from the vagus nerve in NF-1 patients are rare [3, 4]. Plexiform neurofibroma of the bilateral cervical vagus nerve is extremely rare. The clinical presentation of these entities is usually not related to the tumor size. Dysphagia, vocal cord paralysis, hoarseness of voice, and heart rate changes are the most common manifestations; however, none of these symptoms existed in the current case.

Preoperative diagnosis of a neurofibroma arising from cervical vagus nerve is challenging. The differential diagnosis includes enlarged lymph node and other neurogenic tumors. Echogenic hilum of a lymph node can serve as an important clue to differentiate it from neurogenic tumors. Only tumors arising from the vagus nerve can splay the internal jugular vein and the carotid artery, and the tumors arising from the carotid body will push the vascular structures to lateral or medial position. Lymph nodes and carotid body tumors would not demonstrate continuity with vagus nerve.

A preoperative diagnosis is critical to surgical planning. The internal carotid artery or internal jugular vein splaying, together with the specific clinical symptoms facilitate the preoperative identification of the involved nerve [5]. Schwannomas are common isolated, unilateral tumors. Internal cystic changes are more common in schwannomas. In addition, isolated schwannomas can be completely resected from the parent nerves. On the contrary, neurofibromas tend to intertwine themselves with several fascicles of nerve of origin. The adjacent nerves may show irregular thickening. Therefore it is often impossible to completely remove the neurofibroma whilst ensuring preservation of the parent nerve. Surgical resection of neurofibromas is therefore more difficult than that of schwannomas.

Neurofibromas typically exhibit intermediate signal intensity on T1-weighted images, and in T2-weighted images these exhibit a moderate to marked high signal intensity, as was seen in the current case. MR imaging can clearly observe the relationship between large tumors and the adjacent structures, but this modality may not delineate a small mass like the neurofibroma of the left vagus nerve in our patient. In such cases, high-resolution US can overcome the shortcomings of MRI. Color Doppler US can detect low-velocity arterial flows within the mass, and explicit the anatomical relationship between the mass and its peripheral vascular. Additionally, it makes it feasible to evaluate adherence of the tumor to vessels by applying gentle pressure on the probe. Moreover, the high-resolution images on US can help to identify the origin of tumors. In the present case, US revealed a hypoechoic tumor centered on the carotid sheath; both the proximal and distal ends of the tumor were in continuity with the vagus nerve; these radiological features were important diagnostic clues to its vagal nerve origin. Once a diagnosis of neurofibroma of the vagus nerve is made on one side, the possibility of bilateral tumors should be considered. This prompted us to scan the other side of the neck as well, which revealed a fusiform mass involving the left vagus nerve. Additionally, we scanned the extremities, and found the sural nerve was also affected. Together with the widespread cafe-au-lait spots, this patient met the diagnostic criteria for NF-1 [2]. That would truly be a lesson learned from the paper - I would have relied on the MR imaging alone for surgery!

In the current case, the right tumor was completely resected from the proximal and distal vagus nerve, and we cut the distal affected vagus nerve as well. Since both vagus nerves were affected; considering the potential risk of bilateral vagal resection such as dysphonia, dysphagia, cough, tongue weakness, hoarseness, cranial nerve palsy, and cardiac events [6], we recommended monitoring of the smaller left-sided neck mass.

## Conclusions

Our experience suggests that US can be a valuable method for preoperative evaluation of a cervical mass, as it is capable of displaying the vagus nerve and provides sufficient diagnostic information. Therefore, high-resolution US could be an alternative diagnostic imaging modality to MR imaging for patients with suspected neurogenic tumors of the cervical vagus nerve, especially in patients for whom MR imaging is contraindicated.

## Abbreviations

MR: Magnetic resonance
NF-1: Neurofibromatosis type 1
US: Ultrasonography
